# Complete chloroplast genome sequences of two *Boehmeria* species (Urticaceae)

**DOI:** 10.1080/23802359.2018.1502635

**Published:** 2018-08-23

**Authors:** Zeng-Yuan Wu, Xin-Yu Du, Richard I. Milne, Jie Liu, De-Zhu Li

**Affiliations:** a Germplasm Bank of Wild Species, Kunming Institute of Botany, Chinese Academy of Sciences, Kunming, People’s Republic of China;; b Institute of Molecular Plant Sciences, School of Biological Sciences, University of Edinburgh, Edinburgh, UK;; c Key Laboratory for Plant and Biodiversity of East Asia, Kunming Institute of Botany, Chinese Academy of Sciences, Kunming, People’s Republic of China

**Keywords:** *Boehmeria*, chloroplast genome, phylogeny, Urticaceae

## Abstract

*Boehmeria* is an important genus; however, no plastid genome has been reported to date. Here we report the complete chloroplast genomes for two *Boehmeria* species. The chloroplast genomes of *Boehmeria umbrosa* and *Boehmeria spicata* were found to be 170920 bp and 170958 in length, respectively, and the GC contents were 35.5 and 35.3%, respectively. The sequences of each species contained 112 unique genes, including 30 tRNA, 4 rRNA, and 78 protein-coding genes. This is the first report of cp genomes for *Boehmeria*, and will be useful for identifying molecular markers with which to address taxonomic problems in the genus.

*Boehmeria* Jacquin (Urticaceae) comprises approximately 47 species and is widely distributed in tropical and temperate regions (Wilmot-Dear and Friis 1996, 2013). It is an economically important genus which provides high-quality fibre (Chen et al. 2003). However, relationships within the genus still remain poorly resolved.

Information from chloroplast genomes has been extensively applied in understanding plant relationship (Ma et al. 2014; Du et al. 2017). To date, however, no complete plastid genome has been reported for any member of the Urticaceae.

In this study, we report and characterize the complete chloroplast genomes of *Boehmeria umbrosa* (Hand.-Mazz.) W. T. Wang and *Boehmeria spicata* (Thunberg) Thunberg, which are endemic to China and East Asia, respectively. Young, fresh, and healthy leaves were collected from *B. umbrosa* on Gaoligong Mountain (Yunnan, China; N 27°46′25.7″ E 98°35′38.35″), and from *B. spicata* on Tianmu Mountain (Zhejiang, China; N 30°20′14.8″, E 119°26′43.9″). Both voucher specimens were deposited in herbarium KUN (collection numbers are GLGE14989 and liuj10748, respectively). Genomic DNA was extracted following CTAB method (Doyle 1987), then sequenced using the Illumina Hiseq 4000. Sequences were assembled by multiple steps, including de novo assembling which was constructed in SPAdes version 3.9.1 (Bankevich et al. 2012), using k-mer lengths of 85–115 bp; then we used reference guided assembling conducted with Bandage version 0.8.1 (Wick et al. 2015) and Geneious version 9.1.4 (Kearse et al. 2012); *Morus notabilis* (NC_027110) was used as reference for assembling and annotation; finally, inverted repeat boundaries were determined by blast, and verified by reads mapping in Geneious version 9.1.4 (Kearse et al. 2012).

The complete chloroplast genome sequence of *B. umbrosa* (GenBank accession number MF990291) was 170920 bp in length, the GC content was 35.5%. LSC and SSC contained 68844 bp and 18462 bp, respectively, while IR was 41807 bp in length. The genome contained 112 functional genes, including 78 protein-coding genes, 30 tRNA genes, and 4 rRNA genes.

The complete chloroplast genome sequence of *B. spicata* (GenBank accession number MF990290) was 170958 bp in length, the GC content was 35.5%. LSC and SSC contained 70994 and 18478 bp, respectively, while IR was 40743 bp in length. The genome contained 112 functional genes, including 78 protein-coding genes, 30 tRNA genes, and 4 rRNA genes.

To identify the phylogenetic positions of *B. spicata* and *B. umbrosa*, a maximum likelihood phylogenetic tree was generated using RAxML–HPC BlackBox (Stamatakis 2014) through Cipres Science Gateway (Miler et al. 2010), based on concatenated complete chloroplast genomes from the two *Boehmeria* species, one *Debregeasia* species (GenBank-KY419997, which is only partial plastid genome) and other 12 species from Moraceae, Ulmaceae, Cannabaceae, and Rosaceae. Consistent with our previous results (Wu et al. 2013), present results showed that two species grouped into one well-supported clade and formed a sister to *Debregeasia* (Figure 1). These newly characterized chloroplast genomes of *Boehmeria* can be used to develop markers for further study on the phylogeny and evolution of the genus *Boehmeria*, and also to clarify species boundaries, which is important for conservation.

**Figure 1.**
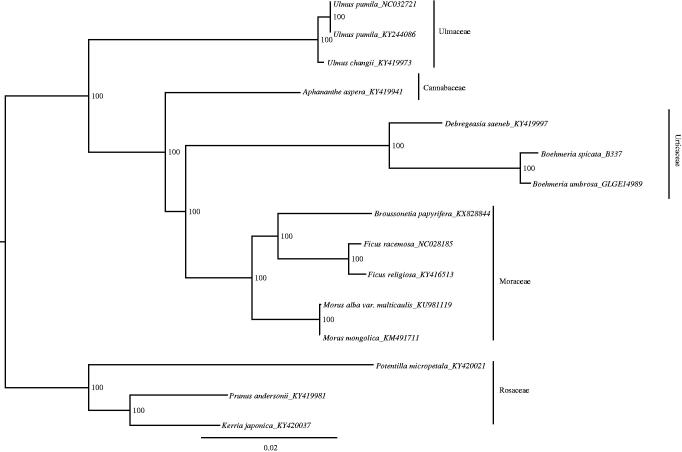
Phylogenetic tree produced by Maximum Likelihood (ML) analysis base on chloroplast genome sequences from 15 species of Rosales, numbers associated with branched are assessed by Maximum Likelihood bootstrap.
